# Evolutionary origins of human handedness: evaluating contrasting hypotheses

**DOI:** 10.1007/s10071-013-0626-y

**Published:** 2013-04-02

**Authors:** Hélène Cochet, Richard W. Byrne

**Affiliations:** Scottish Primate Research Group, Centre for Social Learning and Cognitive Evolution, School of Psychology and Neuroscience, University of St Andrews, St Mary Squad, South street, St Andrews, Fife, KY16 9JP UK

**Keywords:** Hand preference, Hemispheric specialization, Communicative gestures, Evolution of language, Nonhuman primates, Human children

## Abstract

Variation in methods and measures, resulting in past dispute over the existence of population handedness in nonhuman great apes, has impeded progress into the origins of human right-handedness and how it relates to the human hallmark of language. Pooling evidence from behavioral studies, neuroimaging and neuroanatomy, we evaluate data on manual and cerebral laterality in humans and other apes engaged in a range of manipulative tasks and in gestural communication. A simplistic human/animal partition is no longer tenable, and we review four (nonexclusive) possible drivers for the origin of population-level right-handedness: skilled manipulative activity, as in tool use; communicative gestures; organizational complexity of action, in particular hierarchical structure; and the role of intentionality in goal-directed action. Fully testing these hypotheses will require developmental and evolutionary evidence as well as modern neuroimaging data.

## Introduction

Although lateralization was present early in vertebrate phylogeny (e.g., MacNeilage et al. 2009; Rogers and Andrew 2002) and is even known in invertebrates (e.g., Frasnelli et al. 2012), the manifestation of cerebral and functional asymmetries in the form of handedness has been argued to distinguish the human species, notably in connection with hemispheric dominance for language (e.g., Corballis 1991; Knecht et al. 2000). However, the nature of the relationship between these asymmetries is still unclear, mainly because *handedness* can embrace multiple dimensions. The existence of a strong right-handed bias in humans may therefore be linked to different extents to the left-hemispheric dominance for language, depending on these different dimensions.

In the present review, we adopt a comparative approach to the origins of laterality in manipulative and communicative behaviors in human and nonhuman primates, in order to investigate the relationship between language and hand preference. We examine evidence from different disciplines such as developmental psychology, neuroscience, archeology, and primatology that may shed some light on the origins of human handedness. This review is arranged in three sections, beginning with a presentation of the different methods and categorizations used by researchers to study hand preferences in human and nonhuman primates. Taking into account these methodological distinctions, we then review the current data on manual and cerebral asymmetries in human and nonhuman primates. This allows us to examine in the third section several different hypotheses about the origins of handedness and hemispheric specialization for language.

In this paper, we will consider manual asymmetries both at the *population* level, to characterize a species’ bias to the left or the right hand (as estimated by the sample studied), and at the *individual* level to describe an individual’s tendency to favor one hand over the other. We will use the terms of *handedness* and *hand preference* to refer to the direction of manual asymmetries for different activities; when we refer to the *strength* of asymmetries, we will say so explicitly.

## Variability in assessment of hand preferences

### General methodological questions

Hand preference in human and nonhuman primates has been described in terms of different categorizations and by using different methods, which has yielded discrepancies about the degree of the right-hand bias in humans and about the existence of population-level asymmetries in nonhuman primates. In order to identify the processes involved in the evolution of manual specialization, we thus need to consider the different approaches taken to the study of hand preference.

Several methodological variables need to be considered, some of which have previously been emphasized in studies of both human and nonhuman apes (e.g., Healey et al. 1986; Marchant and McGrew 1991). The characteristics of the population studied are among the first variables of importance, for example, in terms of age. The degree of right-handedness strengthens with age in the course of human development (e.g., McManus et al. 1988), and nonhuman primates tend to exhibit greater strength of hand preference as adults than as immatures (e.g., in chimpanzees: Humle and Matsuzawa 2009). Sex can also influence hand-preference patterns: meta-analyses in human adults have shown a higher prevalence of left-handedness in males than in females (Sommer and Kahn 2009). A comparable effect of sex on manual asymmetries has been reported in nonhuman primates (e.g., in chimpanzees: Corp and Byrne 2004; in squirrel monkeys: Meguerditchian et al. 2012), though apparently weaker than in humans (e.g., Meguerditchian et al. 2011).

Moreover, laterality data can be collected in very different settings and conditions. In nonhuman primates, experiments to induce behaviors in captive individuals are generally contrasted with the observation of spontaneous behaviors in wild individuals, though experimental manipulations can also be used in natural conditions. All these different approaches have their advantages and disadvantages. Right-handedness in populations of captive apes has, for example, been argued to be a by-product of exposure to human culture (McGrew and Marchant 1997); but the sample sizes are often larger in studies of captive apes than in studies of wild apes, which increases the possibility of detecting significant population-level asymmetries (Hopkins et al. 2012a, b). Experimental studies in captive subjects also allow researchers to gain some control over the effect of postural and positional biases on hand use, thus reducing the noise in assessing hand preferences.

In humans, also, the use of experimental situations and self-report questionnaires in adults can simplify the study of handedness, compared to the observation of manual asymmetries in more natural situations. A more reliable overview of hand preferences may be given by spontaneous behavior because it reflects an immediate motor component, unlike data collected with questionnaires (e.g., Cavill and Bryden 2003), but such studies are time-consuming. More recently, researchers have used experimental tasks in ecologically relevant contexts, namely contexts in which object use is necessary to reach a specific goal rather than those in which participants are directly asked to use a particular object (e.g., Cochet and Vauclair 2012). These different conditions lead to wide variations in sample sizes, which contribute to explaining the differences across studies in the reliability with which handedness is shown at the group level.

Another issue in the study of manual asymmetries relates to the definition of handedness: Some researchers focus only on the direction of manual asymmetries, with a simple left–right dichotomy, whereas others also use intermediate categories to measure less consistent preferences. The classification of individuals usually involves statistical analyses, but it can also depend on thresholds that are defined a priori using the number or proportion of left- and right-hand responses. Moreover, researchers do not always use categories, sometimes focusing on the continuously distributed strength of hand preference (see Hopkins 1999). The same issues arise at the population level, as definitions of left- or right-handedness, based on the number of lateralized individuals, do not always rely on statistical analyses.

Finally, the number of responses used to assess individual hand preferences is also a source of variation between studies, which has been argued to influence the apparent strength of the effects. However, the direction of this influence is still unclear: When the number of observations per individual increases, the number of ambidextrous individuals has sometimes been found to increase (Palmer 2002) or to decrease (Meguerditchian et al. 2011). At least until we elucidate these contradictory findings, it seems safer to standardize the number of responses across individuals and across tasks in studies of hand preference.

### Manual asymmetries for different activities

Beyond differences in the sample characteristics and the general methods of data collection, there are important disparities between studies in the nature of the activities chosen to record hand preferences. These activities can be categorized in different ways, depending on the specificity of research questions. The existence of manual asymmetries has traditionally been highlighted by focusing on manipulative activities, in part because object-directed asymmetries are more salient and easier to assess than asymmetries for empty-handed activities. A further distinction has been made within the category of object manipulations based on the complexity of the activity, since skill levels may influence the strength of individual hand preferences, and this can also be reflected at the population level. For instance, some activities require fine motor skills and coordination between the dominant hand that plays an active role and the nondominant hand that has a role of support or orientation. In both human and nonhuman primates, activities involving this ‘asymmetric bimanual coordination’ are associated with stronger and more stable individual hand preferences than unimanual activities, such as object grasping, as well as with a greater degree of right-handedness at the population level (e.g., Byrne and Byrne 1991; Fagard and Lockman 2005).

In recent years, research has also begun into hand preferences for making communicative gestures. Although there are still relatively few data regarding asymmetries in gesturing, researchers have focused on several different types of gestures: from intra-specific gestures and gestures directed to humans in nonhuman primates (e.g., Hobaiter and Byrne 2013; Hopkins and Wesley 2002; Meguerditchian and Vauclair 2006) to co-speech gestures and pointing in humans (e.g., Meunier et al. 2012; Saucier and Elias 2001). Possible variation in asymmetry with different functions of pointing gestures has also been taken into account in studies with young children (Cochet and Vauclair 2010). Several distinctions can thus be made, within the category of communicative gestures, which might influence hand-preference patterns (see ‘Variability in assessment of hand preferences’).

The study of manual asymmetries for different activities has led to a functional categorization contrasting communicative and noncommunicative activities: the former referring essentially to empty-handed gestures (e.g., Rowe and Goldin-Meadow 2009) and the latter to object manipulations (e.g., Fagard and Marks 2000). Because some activities can be both communicative and manipulative, it might also be useful to add a third category, namely communicative gestures involving objects (Hobaiter and Byrne 2013). Cutting across this communicative/noncommunicative categorization is another, based on the nature of the target: Some differences have been found between actions given toward animate and inanimate objects (in gorilla: Forrester et al. 2011; in chimpanzee: Forrester et al. 2012). Here, ‘actions toward animate objects’ refers to actions performed toward both the self and conspecifics, and not necessarily involving any communicative goals; finding correspondence between these different categorizations is therefore not straightforward.

Thus, descriptions of handedness include numerous features, making the comparison between studies, and especially between species, more complex. In the following section, we consider some of these features when presenting recent data on manual asymmetries and report also some neuroimaging evidence for cerebral asymmetries in human and nonhuman apes.

## Manual asymmetries in human and nonhuman primates

### Nonhuman apes: behavioral and cerebral asymmetries

Although there is no doubt that some individuals show strong individual hand preferences, differences in the methods used to study manual asymmetries—notably in terms of sample, task, and context—have resulted in discrepant findings about the existence of species-level handedness in nonhuman primates. In groups of captive individuals, right-handedness has been demonstrated in skilled tasks that require coordinated bimanual actions (e.g., Hopkins 2006), whereas such a population-level bias has not been observed for simple unimanual tasks, including, for example, object grasping (e.g., Vauclair et al. 2005). In wild individuals, the existence of handedness has been more debated, and this question is sometimes difficult to address due to limited sample size. Several studies have failed to show any significant population bias in wild chimpanzees (e.g., Corp and Byrne 2004; Humle and Matsuzawa 2009; McGrew and Marchant 2001). However, the use of different methods, including different tasks (see Hopkins and Cantalupo 2005), has revealed small but statistically significant population-level biases for some bimanual or otherwise complex actions. In different species and tasks, these biases range from 58 to 66 % lateralized in one direction: all lower than the 90 % typically quoted for human right-handedness (although this percentage can vary depending on the method used, see below). For example, right-handedness was found for three types of bimanually coordinated leaf-gathering in mountain gorillas (Byrne and Byrne 1991) and for nut-cracking in chimpanzees (Lonsdorf and Hopkins 2005), whereas left-handedness was found for termite fishing in chimpanzees (Lonsdorf and Hopkins 2005) and for an experimentally introduced bimanual tube task in snub-nosed monkeys (e.g., Zhao et al. 2012). Notice that in chimpanzees and mountain gorillas, handedness was task specific: In the latter species, subjects showed equally strong individual laterality for processing leaves and stems, but population biases were quite different (Byrne and Byrne 1991).

In addition, right-handedness has been reported for some communicative gestures produced by nonhuman primates, including gestures used to request food from a human partner (e.g., Meguerditchian et al. 2010); gestures used in captivity to threaten and intimidate conspecifics (e.g., Meguerditchian et al. 2011); and in the wild for communicative gestures employing objects (Hobaiter and Byrne 2013). The distinction between communicative and noncommunicative activities has highlighted a stronger right-handed bias for gestures than for manipulative activities, as well as the absence of significant correlation between the two types of asymmetry (Meguerditchian and Vauclair 2009; Meguerditchian et al. 2010). Some studies have also shown the absence of any correlation between individual handedness for different manipulative activities (e.g., Byrne and Byrne 1991), whereas the manual asymmetries reported for different communicative gestures are significantly correlated with each other (in chimpanzees: Meguerditchian et al. 2010; in human infants: Cochet and Vauclair 2010). These results suggest that researchers need to go beyond the distinction between communicative and manipulative activities to understand the origins of hand preference. Comparing the nature of the target has shown a significant right-handed asymmetry in gorillas and chimpanzees for actions toward inanimate objects, but not for those toward animate ones (Forrester et al. 2011, 2012), thus offering an alternative direction to investigate the functional causes and the evolution of manual specialization (see ‘Manual asymmetries in human and nonhuman primates’).

Neuroimaging data from chimpanzees have provided further support for the existence of hemispheric asymmetries in nonhuman primates. Leftward anatomical asymmetries, for example, in the proportion of white matter in the motor hand area (a characteristic knob of the precentral gyrus) and in homologues to language areas in humans, seem to be associated with right-handed asymmetries in some activities, such as throwing (Hopkins et al. 2012a, b) and coordinated bimanual actions (Gilissen and Hopkins 2013). However, other studies have failed to reveal any significant relationship between neuroanatomical asymmetries in the region of the inferior frontal gyrus, which is regarded as the homologue of Broca’s area, and hand preference for reaching actions (Taglialatela et al. 2006); or they have shown only weak correlations with hand preference for more complex manipulative actions, such as termite fishing (Hopkins et al. 2007). By contrast, in both these studies, neuroanatomical asymmetries were found to be strongly associated with the right-sided bias for communicative gestures.

### Humans: developmental studies and neuroimaging data

A right-sided asymmetry in hand-use patterns for manipulative activities is observed in around 90 % of literate human adults (e.g., Annett 1985; Raymond and Pontier 2004), though lower levels have been found in traditional societies (between 73 and 97 %: Faurie and Raymond 2005; 55 % right-hand use overall, rising to 84 % when only tool use is examined: Marchant et al. 1995). Signs of manual asymmetries in object manipulation are manifested early in infancy (see Provins 1992), but the degree of right-hand asymmetry stabilizes only in mid-childhood (McManus et al. 1988), after some fluctuations in the early years that have been regarded as successive reorganizations of the motor system (e.g., Corbetta and Thelen 1999; Ferre et al. 2010). As in nonhuman primates, the distinction between unimanual and bimanual activities in human children has revealed a stronger and more stable bias for bimanual coordinated actions (e.g., Fagard and Lockman 2005).

In addition to hand preference for manipulative activities, researchers have investigated the asymmetry of gestures: mainly co-speech gestures in adults (e.g., Kimura 1973; Kita et al. 2007) and communicative gestures such as pointing in children (e.g., Bates et al. 1986; Blake et al. 1994). In human infants and children, the comparison between communicative gestures and noncommunicative activities has highlighted a stronger right-hand bias for pointing gestures than for manipulative activities (Jacquet et al. 2012). Similar to the results in nonhuman primates, hand-preference scores associated with these different activities are not correlated (Cochet and Vauclair 2010; Esseily et al. 2011).

To examine the developmental continuity in these different hand-preference patterns, we have to consider comparable tasks in children and adults. However, whereas the use of self-report questionnaires and complex experimental tasks is widespread in adults (e.g., Johansson et al. 2006), ethological activities have seldom been coded (Marchant et al. 1995). A recent study has assessed hand preferences in natural situations, through tasks eliciting familiar object manipulations and pointing gestures (Cochet and Vauclair 2012). Results revealed (1) significant but moderate correlations between hand-preference scores for pointing gestures and bimanual manipulation, and (2) no significant difference between communicative gestures and manipulative actions in the mean strength of hand preference. This study also showed that the strength of right-handedness for manipulative activities was much greater in adults than that reported in young children, whereas the difference with age was rather slight for pointing gestures. This comparison suggests that the emergence of hand preference in the course of human ontogeny may be driven by communicative gestures, and the later strengthening of right-hand preference for object manipulations may relate to an increasing need to use complex tools (Cochet and Vauclair 2012). In addition to changes in individual lateral bias with age, population-level right-handedness may also derive from communicative gesturing during development, although empirical data are still needed to further support this hypothesis.

Moreover, neuroanatomical data have emphasized the existence of strong leftward structural asymmetries in the human brain (e.g., in the relative white matter content), especially in language-related regions of the frontal and temporal regions (Pujol et al. 2002). Researchers have long tried to draw a parallel between left-hemispheric dominance for language and the strong population-level right-hand bias for object manipulation in humans. However, it has been shown that the direction of handedness for manipulative actions is not a good indicator of hemispheric dominance for speech: The left cerebral hemisphere is dominant for language in right-handers (96 %, Knecht et al. 2000; Pujol et al. 1999), but also in majority of left-handers (73 %, Knecht et al. 2000; 76 %, Pujol et al. 1999). By contrast, there may be a more direct relationship between handedness for gestures and hemispheric dominance for language (Kimura 1973). Neuroimaging studies have indeed demonstrated that gestures and speech are controlled by common networks in left-lateralized inferior frontal and posterior temporal regions (e.g., Willems et al. 2007; Xu et al. 2009). A study using event-related brain potentials has also shown that semantic information conveyed through speech and gestures is integrated simultaneously by the brain (Özyürek et al. 2007). These studies, providing some insight into the processes of hemispheric lateralization, have thus highlighted the close relationship between gestures and language.

Other studies have reported the existence of neurons controlling grasping movements of both hand and mouth (see Gentilucci and Dalla Volta 2007, for a review), and variations in the size of a grasped object have been found to influence lip-opening kinematics and voice parameters (e.g., Gentilucci et al. 2001). From these findings, it has been argued that gestural laterality is simply a reflection of lateral bias in all actions (e.g., Willems and Hagoort 2007), but complex processes may underlie the relationship between language, action, and gesture, which still deserve to be investigated (see ‘Manual asymmetries in human and nonhuman primates’).

### Laterality studies: comparison between human and nonhuman primates

In part due to the issues we have described, the comparison of hand-preference patterns between human and nonhuman primates requires some theoretical and methodological precautions. First, we must compare tasks that are similar, which may be easier for noncommunicative activities than for communicative activities. Bimanual coordination activities can, for example, be observed with the same task in different ape species, including human infants and adults (e.g., Cochet and Vauclair 2012; Hopkins et al. 2011; Meunier and Vauclair 2007). Between-species comparison may be more intricate for communicative activities because the gestures produced by human and nonhuman primates may not share the same properties, for example, in terms of communicative functions (e.g., Pika 2008), as illustrated by the case of triadic gestures, namely gestures that refer to an external entity for the benefit of another agent. The production of triadic gestures has been argued to be a hallmark of human communication (Camaioni 1997), although the use of such gestures has been reported in captive chimpanzees (Leavens and Hopkins 1998). Moreover, the qualitative nature of gestures may differ between both species, insofar as nonhuman primates’ gestures may not involve the same capacities of attributing mental states to communicative partners (e.g., Grice 1989; Sperber and Wilson 2002; Tomasello et al. 2003 for a review in chimpanzees). The age of emergence of these capacities in the course of human development is still subject to debate (D’Entremont and Seamans 2007; Liszkowski 2011) and so is the comparison between nonhuman primates and human infants (e.g., Leavens and Racine 2009).

Another important point of contrast pertains to the number of responses required to measure handedness. Sample-size effects may be observed at the individual level depending on the number of responses per subject and at the population level depending on the number of subjects included in the study. Whereas studies with human adults and captive nonhuman primates are able to assemble statistically reliable samples, hand preference in children is assessed from a limited number of responses per subject, varying between 2 and 10 across studies (e.g., Cochet 2012; Fagard and Marks 2000; Vauclair and Imbault 2009). This may be explained by the difficulty of maintaining children’s attention over long periods of time. However, to reliably compare hand-preference patterns, it is first necessary to record a similar number of responses for all participants and across different tasks, and this factor may be at least as important as the number of responses per subject. Studies of nonhuman primates have their own difficulties: For instance, it is seldom possible to obtain data from a great number of apes in the wild, and few studies have involved longitudinal designs.

**Fig. 1:**
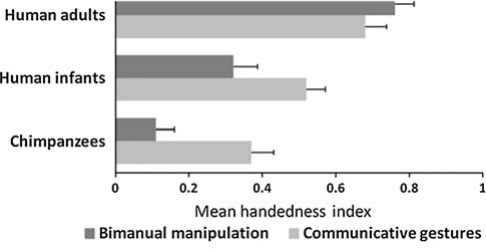
Adapted from Vauclair and Cochet (2013). Mean handedness indices for communicative gestures and bimanual manipulation in chimpanzees (Meguerditchian et al. 2010), human infants (Vauclair and Imbault 2009), and human adults (Cochet and Vauclair 2012). The handedness index is calculated using the formula (R − L)/(R + L), where R and L represent the total number of right- and left-hand responses. It varies from −1 to 1, the positive sign reflecting right-hand preference and the absolute values hand-preference strength

Finally, the comparison between human and nonhuman primates requires the use of the same metric of hand preference. In this perspective, handedness indices provide a more complete measure of manual asymmetries than the categorization as left-hander or right-hander, as they indicate both the strength and the direction of hand preference (see Fig. 1 for an example).

With the above-mentioned issues in mind, we can draw a parallel between the ontogenetic and phylogenetic processes involved in hemispheric lateralization by considering handedness patterns in infants, adults, and nonhuman apes. Population-level right-handed bias is higher in humans than in nonhuman great apes, revealing a stronger degree of specialization, but the analysis of different activities has shown this difference to be greater for manipulative activities than for communicative gestures (see Fig. 1).

Such between-group comparisons, taking into account several activities, can provide some insight into the function, development, and evolution of manual specialization. Given the dominance of the left cerebral hemisphere for language processing in humans, they may also clarify the nature of the relationship between gestures and language. The following section thus presents several hypotheses that attempt to explain the origins of handedness.

## Different hypotheses about the origins of handedness

In the last decades, much effort has been devoted to determining the origins of human handedness, leading to a description of genetic, hormonal, and environmental factors whose interaction would cause neural and behavioral asymmetries. Genetic models have been proposed, postulating that a single gene in the human species determines first language lateralization and second hand preference (e.g., Annett’s right shift theory 1985; McManus 1999). However, the conformity of these models to molecular and behavioral data has been questioned (e.g., Corballis et al. 2012; McManus 2002), and the influence of epigenetic factors on lateralization processes has been highlighted in humans and other animals (e.g., Chiandetti and Vallortigara 2009; Schaafsma et al. 2009). For example, prenatal lateralized motor behaviors, such as thumb sucking and head position, have been shown to influence the subsequent development of hand preference in humans (e.g., Hepper et al. 2005; Ververs et al. 1994), and more complex environmental and cultural factors can come into play as well (e.g., Fagard and Dahmen 2004; Vuoksimaa et al. 2009). It should also be noted that recent research has suggested new directions, with some animal models now including the levels of genes, neurons, and behavior (e.g., in zebrafish: Roussigne et al. 2012).

However, none of these causal descriptions specify to what extent handedness is related to the left-hemispheric dominance for language and/or to the left-hemispheric dominance for the planning of motor actions. Here, we therefore describe several hypotheses that may explain the emergence of right-hand preference and left-hemisphere specialization from a more functional point of view, both at the ontogenetic and at the phylogenetic levels.

### Manipulative activities: lateralization driven by tool use

In humans, anatomical differences between the two cerebral hemispheres result in a greater connectivity of the left motor cortex, which is associated with some superiority in trajectory control and visual feedback for movement (Goble and Brown 2008). Behavioral evidence has shown that the left cerebral hemisphere is dominant for the planning of motor actions in both right-handers and left-handers (Janssen et al. 2011). Because it is a striking example of motor planning, involving structural sequences of events produced to reach a specific goal, tool use has been regarded as the foundation of left-hemispheric lateralization (e.g., Frost 1980). Archeological data revealing prehistoric hand-use patterns for tool use and cave art have confirmed that right-handedness was already established in Neanderthals (Cashmore et al. 2008) and that it may have emerged through the increasing frequency of complex, bimanually differentiated, tool-using activities (Uomini 2009). The observation in gorillas and chimpanzees of a significant right-handed asymmetry for actions toward inanimate targets, but not for actions toward animate ones, provides further support for the hypothesis that right-handedness has emerged from primitive manipulative activities (Forrester et al. 2011, 2012). Moreover, imaging studies have shown that tool use and language perception in humans involve common neural processes in Broca’s area (Higuchi et al. 2009).

However, this ‘tool-use hypothesis’ does not account for the fact that handedness for tool use is not directly related to hemispheric dominance for language, since majority of left-handers do not exhibit a right-hemispheric dominance for language (Knecht et al. 2000; Pujol et al. 1999; Tzourio et al. 1998). In addition, this theory would predict that we should observe, at the population level, stronger right-handedness for manipulative activities than for any other activities, whereas communicative gestures have been reported to be more right-handed than tool use in infants and in nonhuman primates (e.g., Jacquet et al. 2012; Meguerditchian et al. 2010). If developmental patterns observed in human children parallel the evolution of language at the phylogenetic level, we can therefore infer that manipulative activities per se were not the key to the emergence of handedness and brain lateralization. By contrast, communicative gestures may have played an important role in the evolution of cerebral asymmetries.

### Communicative activities: laterality driven by gesture use

The relative rates of growth of the two cerebral hemispheres in humans, mentioned as part of the tool-use theory, have also been invoked to argue for a primary role for gesture. Here, the development of generative skills between 2 and 4 years, which are crucial to the development of language (Corballis 1991; Studdert-Kennedy 1998), correlates with brain growth principally in the left hemisphere. Thus, merely through the typical growth gradient in the brain, the emergence of manual asymmetries may correlate with the development of communicative skills in early stages. The strong degree of right-hand preference reported for communicative gestures in infants and toddlers, and in particular for pointing gestures serving complex functions (Cochet and Vauclair 2010), supports this hypothesis. The right-handed asymmetry observed for some intentional gestures produced by nonhuman primates (Hopkins et al. 2012a, b) also suggests that gestural communication has played a key role in the evolution of hand preferences and cerebral asymmetries at the phylogenetic level (Corballis 2012).

Moreover, the relationship between right-handedness and left-hemispheric specialization for language seems to be driven by a need for laterality in gestural communication, only secondarily reflected in noncommunicative activities (e.g., Kimura 1973). The close interconnection between language and gesture has also been emphasized by studies demonstrating the influence of gestures on voice parameters: For instance, voice pitch increases when a word and the corresponding gesture are produced simultaneously, compared to conditions involving only the production of words or involving both modalities but meaningless arm movements and pseudo-words (Barbieri et al. 2009; Bernardis and Gentilucci 2006). Imaging studies have revealed that the perception of language and communicative gestures activates common neural networks in the left cerebral hemisphere (e.g., Xu et al. 2009). The existence of a modality-independent communication system in the left cerebral hemisphere has been interpreted within a framework about language origins and has led some researchers to assign gestures a key role in the evolution of communication and hemispheric specialization (e.g., Corballis 2003; Vauclair and Cochet 2013). Whether the evolutionary precursors of human language involve first and foremost gestures (e.g., Hewes 1973) or a combination of gestures and vocalizations (e.g., Hopkins and Cantero 2003; Masataka 2008), both developmental data and primate studies have shown the importance of gestural communication in social interactions (e.g., Goldin-Meadow 2007; Hobaiter and Byrne 2011b).

### Organization of action: laterality driven by hierarchical structure

As we have previously noted, some authors consider that laterality in manual skills or communicative gestures are merely a subset of a more pervasive lateral bias in all actions (e.g., Willems and Hagoort 2007). However, clearly not all actions show equal degree of asymmetry; one explanation for this is that the extent of bias shown depends on the complexity of action organization. The left cerebral hemisphere appears to be specialized for processing hierarchical structures, whether the latter express themselves through manipulative activities, gestures, or language (e.g., Hauser et al. 2002; Sperry 1982). Therefore, the relationship between language dominance and hand preference might be apparent only when the activities involve a certain level of complexity in terms of organization and execution. Tool-use skills and language both involve a sequential organization, which manifests itself, respectively, through manual movements and words (or signs), with the emergence of grammatical abilities (Forrester and Quaresmini 2013). Moreover, the common neural responses elicited by tool use and language perception in humans (Higuchi et al. 2009) have suggested that Broca’s area may be involved in the processing of structured sequences of elements.

This ‘hierarchical structure hypothesis’ can explain some discrepancies observed in studies with human adults: Significant correlations have been reported between hemispheric specialization for language and hand preference for manipulative activities, such as flipping a coin and striking a match, but not for other activities (Bryden et al. 1994).

In nonhuman great apes, right-handedness might also apply more specifically to activities involving actions that are employed in a structured way, such as leaf-gathering (in gorillas: Byrne and Byrne 1993), or that are employed hierarchically, such as tool use in nut-cracking (in chimpanzees: Lonsdorf and Hopkins 2005). However, hand preferences for leaf-gathering were shown not to be correlated with those for stem processing in wild gorillas (e.g., Byrne and Byrne 1991), although both activities are hierarchically organized, and termite fishing has been characterized by left-handedness in a population of wild chimpanzees (Lonsdorf and Hopkins 2005), results which seem flatly to contradict the hierarchical structure hypothesis. However, left-hand preference might reflect the asymmetry in favor of the right cerebral hemisphere for haptic sensory processing (LaCreuse et al. 1999; Spinozzi and Cacchiarelli 2000), which is required in termite fishing since the chimpanzees do not have any visual feedback of the quantity of termites accumulated before extracting the stick, overruling any tendency toward right-handedness from the task’s organizational needs.

Finally, it is not always possible to describe precisely different types of activities in terms of hierarchical structure, especially as it is sometimes difficult to identify the dominant hand in activities when both hands work in a complementary way (e.g., Boesch 1991). Moreover, there is no evidence in great apes of any sequential organization for actions other than tool use. In some contexts, chimpanzees use series of gestures (e.g., Hobaiter and Byrne 2011a), but the latter do not involve any hierarchical structure. This hypothesis therefore needs further empirical support.

### Goal directedness: laterality driven by intentionality

Another potential key to the emergence of handedness and brain lateralization might be the fact that manipulative activities, communicative gestures, and language are all goal-directed actions. The development of intentionality in ontogeny and phylogeny might thus be linked to the 2–4 year spurt of left-hemispheric growth and specialization. Although this ‘intentionality hypothesis’ also needs further investigation, it is consistent with the view that pantomimes, regarded as communicative actions, are thought to have had a pivotal function in language evolution (Donald 1991; Kendon 2009; Zlatev 2008). Pantomimes represent specific actions using manual and facial gestures and involve clear purposes in communicative contexts. These characteristics may explain the existence of a close relationship between hand preferences for pantomimes and language dominance in human adults (Meador et al. 1999). Moreover, the processing of communicative intentions has been shown to engage a common neural network independently of the modality: that is, for both speech and gestures (Enrici et al. 2011). The right-handed bias reported in chimpanzees for throwing (Hopkins et al. 2012a, b) might suggest that intentionality has played a role in the left-hemisphere specialization associated with language, especially as individuals that reliably throw were found to show significantly better communication abilities than chimpanzees that do not. Hopkins et al. suggest that the motor skills associated with throwing have enabled a greater cortical connectivity between primary motor cortex and the Broca’s area homologue during hominid evolution.

## Limitations

Predictions from the four hypotheses mentioned above may be difficult to test because the different categories of activities that have been used so far to assess hand preferences (see ‘Introduction’) do not necessarily match a distinction based on purpose, hierarchical structure, or intentionality. For instance, manual actions produced toward animate objects were not found to be significantly right-handed in the study by Forrester et al. (2011, 2012), but this category included all types of movements, especially self-directed movements which are not necessarily communicative and sometimes not intentional.

Moreover, although communicative behaviors and noncommunicative manipulative activities do share some surface properties, they may represent two distinct facets of brain lateralization (e.g., Liu et al. 2009). Over the course of evolution, human ancestors may have evolved right-handedness for manipulative activities and right-handedness for gestures for separate adaptive reasons, even if all occurred over a similar timescale. In human development, hand preferences and hemispheric specialization for language may likewise emerge from different processes, even if they are not independent phenomena in adults (Cochet and Vauclair 2012). Also, the development of manual asymmetries is associated with a considerable degree of intra- and inter-individual variability: We cannot exclude the possibility that manipulative activities and communicative gestures provide different contributions to the development of hand preference depending on the individual and/or the culture.

## Conclusion

In this review, we have adopted a comparative approach to the origins of cerebral specialization by focusing on communicative behaviors, including language, and manipulative activities. The analysis of manual asymmetries in human and nonhuman primates has provided some answers to the question of whether or not there is a common substrate for language and handedness. So far, there is some evidence that tool use served as a preadaptation for left-hemisphere specialization for language, as well as evidence supporting the role of communicative gestures in this specialization. Moreover, if we focus on manipulative activities or communicative gestures, hand preference happens to vary widely depending on the task performed. It is thus likely that hemispheric dominance for language is actually associated with some specific characteristics common to just those tasks eliciting a strong degree of right-handedness. A growing body of work suggests that features of intentionality and hierarchical structure may explain the functional origin of cerebral and manual asymmetries. The further description of these features will clarify the processes involved in the evolution and development of handedness and may also reconcile the defenders of the different theories.

It still appears necessary to examine data from several disciplines, in particular developmental psychology and primatology, using similar definitions and methods and paying critical attention to the task used. Considering evidence in other species of vertebrates may also bring a broader picture of the evolution of lateralization (for reviews: Bradshaw and Rogers 1993; Csermely and Regolin 2012) and thus help decipher the processes underlying cerebral asymmetries in humans.
